# CdSe/ZnS Quantum Dots Impaired the First Two Generations of Placenta Growth in an Animal Model, Based on the Shh Signaling Pathway

**DOI:** 10.3390/nano9020257

**Published:** 2019-02-14

**Authors:** Wuding Hong, Huijuan Kuang, Xingping He, Lin Yang, Pengfei Yang, Bolu Chen, Zoraida P. Aguilar, Hengyi Xu

**Affiliations:** 1 State Key Laboratory of Food Science and Technology, Nanchang University, Nanchang 330047, China; 407205116020@email.ncu.edu.cn (W.H.); huijuankuang@126.com (H.K.); hxpoutlook@126.com (X.H.); ylwyh7729836@126.com (L.Y.); hnayypf@126.com (P.Y.); 407205117055@email.ncu.edu.cn (B.C.); 2 Zystein, LLC., Fayetteville, AR 72704, USA; zaguilar@zystein.com

**Keywords:** CdSe/ZnS QDs, multigenerational effects, placenta, Shh signal pathway

## Abstract

The toxicity, especially the transgenerational toxicity of quantum dots (QDs) in vivo, is still scarcely understood in spite of great promising applications of QDs in biomedicine. In this study, the maternal status, pregnancy outcome, and fetus development of parental generation (P0) to offspring in three generations (F3) were investigated after Kunming mice perinatal (GD 13-PND 5) exposure to Cd containing QDs (CdSe/ZnS QDs) and CdCl_2_. The results show CdSe/ZnS QDs induced placenta injuries in P0 and diminished placenta diameters in F1 and F2. Bodyweight growth decreased in the CdSe/ZnS QDs treatment group in the F1 and F2 generation. Additionally, CdSe/ZnS QDs significantly altered the expression of key genes in the Shh signal pathway. Overall, this study exhibited that the CdSe/ZnS QDs exposure during perinatal period impaired placenta growth in the first two generations, but not on the third generation. The toxicological actions of the CdSe/ZnS QDs might be through the effects on the Shh signal pathway.

## 1. Introduction

Quantum dots (QDs) are classified as semiconductor inorganic nanoparticles, which are synthesized with elements that come from group III-V elements (e.g., InP and InAs) or group II-VI elements (e.g., CdSe and CdTe). Due to the nanoscale size and excellent fluorescence properties, QDs can be applied for tumor diagnosis, drug targeting, biomedical imaging, and other biomedical applications [1,2,3,4]. However, QDs could induce potential toxicity because of the elemental composition and nanoscale size during medical applications or accidental exposures. Tang et al. reported that intravenous exposure to QDs triggered liver injury in mice at a dosage of 10 nmol/kg body weight (BW) [5]. CdSe QDs with a surface coating of ZnS at a concentration of 0.23 pmol/cell caused Xenopus embryonic cell injury observed by Dubertret et al. [6]. Moreover, there are several in vitro and in vivo studies aimed to explore the toxic effects and mechanisms of QDs [7,8].

Recently, reproductive toxic effects of QDs had drawn scientific concerns. Zhang et al. found QDs that accumulated in the placenta and disturbed placental function, inducing severe malformations of the placenta and fetus after exposure to QDs in late pregnancy [9]. Chu et al. reported that QDs could translocate across the placental barrier into the fetus in a dose dependent manner [10]. However, multigenerational toxicity as a field of reproductive toxicity of QDs has not been well studied. As far as we know, only one recent study indicated that exposure to QDs for two weeks before mating, at a dosage of 0.81 mg Cd/kg, did not disturb the reproductive outcome and the offspring development over three generations [11]. These findings could result from the utilization of core/shell structure and phospholipid encapsulation that inhibited QDs degradation. Inhibition of QDs degradation reduced the amount of cadmium ions, preventing damage to the placenta during the term of pregnancy. However, there is scarce research focus on multigenerational toxicity of QDs, not to mention multigenerational toxicity induced by perinatal exposure. 

This study was; therefore, aimed to evaluate the multigenerational effects and mechanism of exposure to CdSe/ZnS QDs during perinatal period (gestation day 13 (GD 13)-postnatal day 5 (PND 5)) in mice. The effects on female reproductive performance and the overall health of their first, second, and third generations of litters were determined. The Cd concentrations in pregnant tissues and the transcription levels of genes related to oxidative stress, apoptosis, and fetus development were also evaluated.

## 2. Materials and Methods

### 2.1. Material and Animal Treatment

CdSe/ZnS QDs used in this research was purchased from Ocean NanoTech, LLC (QSH560, Lot#041802#, Springdale, AR, USA). The optical properties of the CdSe/ZnS QDs were characterized by fluorescence and absorption spectroscopy. Transmission electron microscopy (TEM, JEOL USA, Inc. Peabody, MA, USA) and dynamic light scattering (DLS, Microtrac Inc., Montgomeryville, PA, USA) was used to measure primary and hydrodynamic diameter of this material. Approximately 8–10-week old, female Kunming mice, were obtained from the experimental animal center of Nanchang University. These were kept at a 12/12-hour light/dark cycle condition, libitum supplemented food and water at a specific time. All processes involving mice used in this experiment were in accordance with the institutional animal care committee guidelines and this study was approved by the Animal Care Review Committee (approval number 0064257), Nanchang University, Jiangxi, China. At the end of the 1-week adaptation, female mice were mated with healthy male mice. The day vaginal plug appeared was designated as GD 0 and these were randomly divided into three groups (n = 8). These were observed daily and weighed for clinical manifestations. The control group, CdCl_2_ group, CdSe/ZnS QDs group were treated with normal saline, 1.25 μmol/L CdCl_2_, 0.125 μmol/L CdSe/ZnS QDs, respectively at 100 μL/mice/day from GD 13 to GD 18 or PND 5 (total 5- or 10-times exposure) using tail vein injection. The dose used in this research was according to our previous work, because a longer exposure period was planned, the amount of QDs per exposure was lower than before [9]. Total Cd concentration were same in CdCl_2_ and QDs group. These mice were designated as the parental generation (P0). Half of these mice were anatomized at GD 18, while the rest were exposed until PND 5 and anatomized at PND 21. Six female mice in F1, at age of 60 d, were randomly chosen and mated with healthy male mice to make the F2 generation. After that, six female mice in F2 were used to obtain F3. Pregnancy parameters in P0, F1, F2, and fetus development, such as body weight, fetus and placenta length, fetus number, malformation, and fetus body weight growth, were recorded. Other offspring that were not used in mating were anatomized at PND 60, and blood, organs, and tissues were collected and studied.

### 2.2. ICP-MS (Inductively Coupled Plasma Mass Spectrometry) Analysis Cd Caontent

Approximately 0.1–0.5 g of spleen, liver, kidney, lungs, ovary, breast, uterus, and fetus were weighed in 100 mL glass beaker. To each was added 10 mL of nitric acid and 2 mL of per chloric acid and digestion were carried out at 240 °C for one hour. The temperature was raised to 280 °C until the samples dried out. After cool down to room temperature, double distilled water was added to a final volume of 25 mL. These were used to analyze Cd ion concentrations by ICP-MS. 

### 2.3. Histopathological Examination 

The livers and placentas were carefully isolated from P0 generation in GD 18 and submerged in 10% formalin solution immediately after harvest. These tissues were sliced to 5 μm thickness and these slices were mounted on glass slides. Histological images were taken using a Nikon Ti optical microscope (Tokyo, Japan) after hematoxylin-eosin (HE) staining.

### 2.4. Hematological Analysis and Serum Biomarkers Assay 

Blood in P0 (GD18 and PND 5) and F1 (PND 60) was collected to make hematological analysis and serum biomarkers assay. Mean corpuscular hemoglobin (MCH), hemoglobin (HGB), platelets (PLT), mean corpuscular hemoglobin concentration (MCHC), red blood cells (RBC), white blood cells (WBC), hematocrit (HCT), and mean corpuscular volume (MCV) were determined. Serum biomarkers such as albumin (ALB), aspartate aminotransferase (AST), total protein (TP), globulin (GLB), and total bilirubin levels (TBIL); the ratio of albumin to globulin (A/G), and alkaline phosphatase (ALP), and alanine aminotransferase (ALT); and the ratio of aspartate aminotransferase to aspartate aminotransferase (AST:ALT ration), Creatinine (Cr), and uric acid (UA) were verified. These indicators were examined at Adicon clinical laboratories, Nanchang, China.

### 2.5. RT-qPCR Analysis

AxyPrep Multisource Total RNA Miniprep Kit (Axygen Scientific, Union City, CA, USA) was used to isolate the total RNA from the placenta of the P0 and F1 generations according to the manufacturer’s protocol. cDNA was synthesized with Takara PrimeScript^TM^ RT reagent kit (Cat#RR047A, Lot#AK2802) using 1 μg total RNA following measurements of total RNA concentration using software Quantity One, (PDI Inc., New York, New York). The qPCR primers were synthesized by Xiangyin Biotechnology China (Hangzhou China) (Table S1). Quantitative PCR (qPCR) was performed using SYBR^®^ Premix Ex Taq^TM^ II (TakaRa Code: DRR820A) following standard protocol with 7900HT Fast real-time System (Applied Biosystems, Foster City, CA, USA). Thermal cycling program involved 1 cycle at 95 °C for 1 min, followed by 40 cycles of 95 °C for 5 s, and then 60 °C for 1 min. *GAPDH* was used as the reference gene. Relative gene expression levels were determined by the critical threshold (Ct) number and calculated using the 2^−ΔΔ*C*t^ method.

### 2.6. Statistical Analysis

All the values in this study were expressed as mean ± standard deviation and one-way analysis of variance (ANOVA) by L.S.D (SPSS v22.0, SPSS, Inc., Chicago, IL, USA) was used for comparison of results among different groups.

## 3. Results

### 3.1. Characterization of CdSe/ZnS QDs

**Figure 1:**
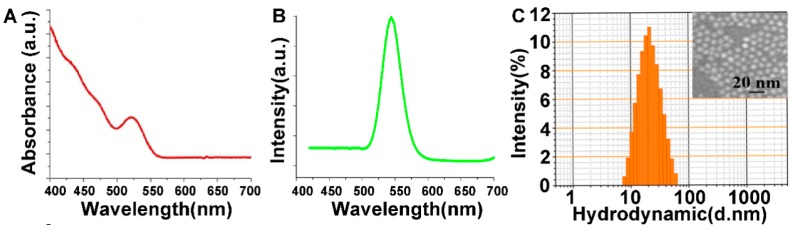
Characterization of CdSe/ZnS quantum dots (QDs). (**A**) absorption; and (**B**) emission spectra of CdSe/ZnS QDs; (**C**) the diameter of CdSe/ZnS QDs determined by dynamic light scattering (DLS) and Transmission electron microscopy (TEM).

The CdSe/ZnS QDs used in this research showed emission peak around 530–550 nm and absorbance peak around 520–540 nm (Figure 1A,B). Dynamic light scattering (DLS) (Figure 1C) analysis indicated that the particles were well resolved in water with an approximate hydrodynamic diameter of 20 nm, which was slighter large than the corresponding size (13 nm) determined by TEM. 

### 3.2. Body Weight Changes of Pregnant Mice

**Figure 2:**
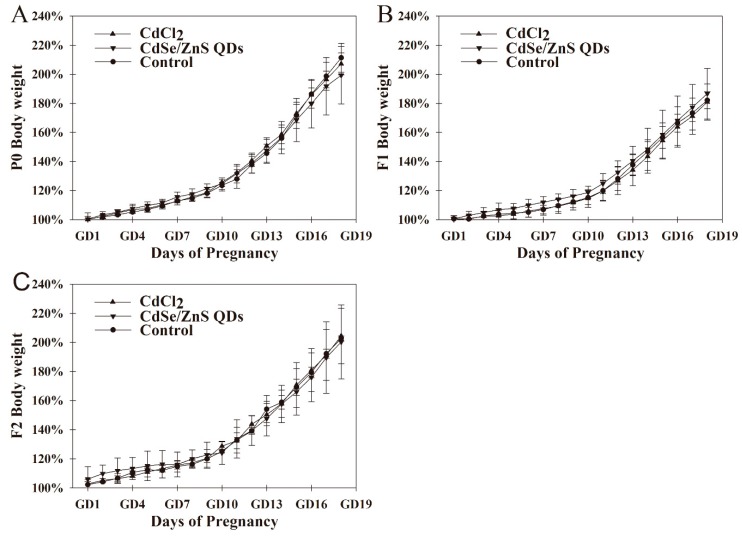
Average body weights of three generational pregnant mice. (**A**–**C**) show changes in maternal body weight of P0, F1, F2, respectively; the body weight of pregnant mice were presented as a percentage of the weight while vaginal plug appeared (GD 0) (= 100%). These data are presented as the mean and SD, n = 6.

In this study, reduction in weight gain were found in P0 pregnant mice after CdSe/ZnS QDs and CdCl_2_ treatment; however, those decreases were not significant (Figure 2A). Furthermore, body weights of F1 and F2 during pregnancy in treatment groups did not exhibit significant changes compared with the control group (Figure 2B,C).

### 3.3. Cd Contents Analysis in Parental Mice (P0)

**Figure 3:**
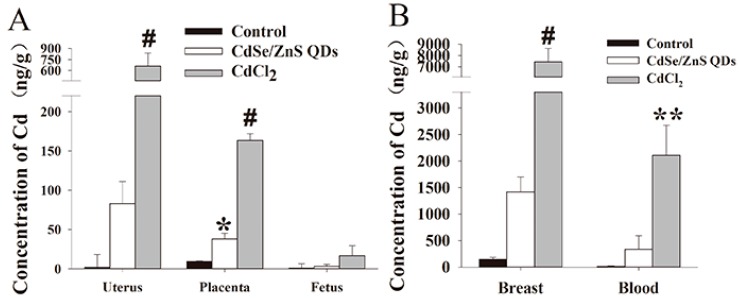
Accumulation of Cd in various organs from different stages of P0 mice. (**A**) uterus, placenta, and fetus in GD 18; (**B**) breast and blood in postnatal day 21 (PND 21). * *p* < 0.05, ** *p* < 0.01, # *p* < 0.001 vs. control. Values were the mean ± standard deviation, n = 5.

In GD 18 (P0), significant elevations of Cd concentrations in placenta were observed in CdSe/ZnS QDs group. (Figure 3) Meanwhile, both uterus and placenta showed obvious amounts of Cd in the CdCl_2_ group. In PND 21 (P0), Cd concentration in breast and blood belonging to the CdCl_2_ group showed an obvious increase compared with the control group.

### 3.4. Hematology and Serum Biochemistry

Before the birthing of P0 (GD 18), mice treated with CdSe/ZnS QDs showed a 3.7% (*p* < 0.05) decrease in MCV level and 49% (*p* < 0.05) decrease in PLT level compared with the control. In addition, the WBC increased by 24% (*p* < 0.05) in the CdCl_2_ treatment group (Table S2). At the end of the suckling period of P0 (PND 21), the levels of RBC and HCT in the mice treated with CdSe/ZnS QDs were statistically lower than the control group at 16% and 18% decrease, respectively. Moreover, the RBC count showed 16% decline, while the MCV level was raised by 8.08% (*p* < 0.05) in the CdCl_2_ treatment group (Table S3). At 60 days old, the F1 female mice in CdCl_2_ and CdSe/ZnS QDs treatment groups showed reductions in MCH by 8.4% and 10.5%, respectively. Meanwhile, the WBC was significantly decreased by 35% in the CdCl_2_ treatment group (Table S4).

**Figure 4:**
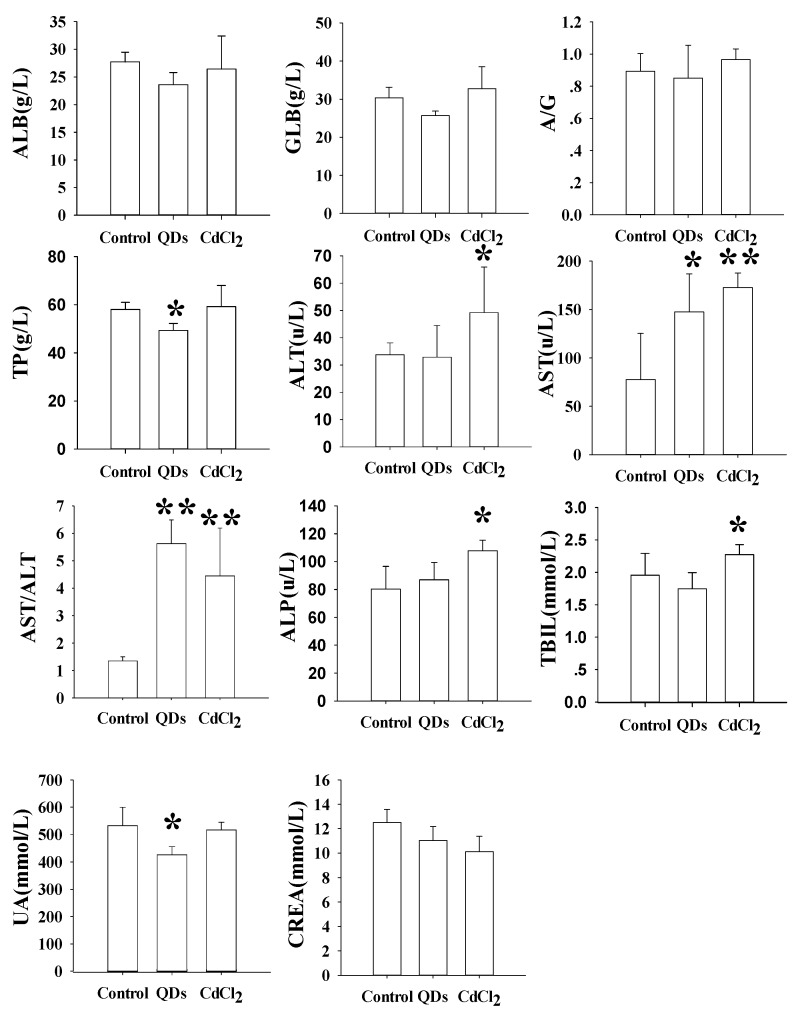
Serum biochemical analysis of F1 mice sacrificed at PND 60. * *p* < 0.05, ** *p* < 0.01 vs. control. albumin (ALB), aspartate aminotransferase (AST), total protein (TP), globulin (GLB), total bilirubin levels (TBIL), the ratio of albumin to globulin (A/G), alkaline phosphatase (ALP), alanine aminotransferase (ALT), the ratio of aspartate aminotransferase to aspartate aminotransferase (AST:ALT ration), Creatinine (Cr), uric acid (UA).

In GD18 (P0), significant increase of AST:ALT ratio (154%, *p* < 0.05) was found in the CdCl_2_ treatment group (Figure S1A). At the end of the suckling period of P0 (PND 21), ALT, AST, and GLB in mice treated with CdCl_2_ showed 48% (*p* < 0.01), 45% (*p* < 0.05), and 15.8% (*p* < 0.05) reduction, respectively, compared with the control group (Figure S1B). The ALB levels in CdCl_2_ and CdSe/ZnS QDs treatment groups showed 13.9% (*p* < 0.01) and 7.5% (*p* < 0.05) decline, respectively (Figure S1B). The Cr levels in mice treated with CdSe/ZnS QDs were statistically lower than the control group with a percent of 26.8% (*p* < 0.05). The TP levels showed 10.4% (*p* < 0.05) and 12% (*p* < 0.01) decrease, respectively in mice treated with CdCl_2_ and CdSe/ZnS QDs. At 60 days of age of F1 female mice, the TP and UA levels significantly decreased by 15% (*p* < 0.05) and 17.9% (*p* < 0.05), respectively, while AST level and AST:ALT ratio significantly increased 90% (*p* < 0.05) and 316% (*p* < 0.01) in the CdSe/ZnS QDs treatment group compared with the control group (Figure 4). Besides, TBIL, AST, ALT, ALT/AST, and the ALP were significantly increased by 14% (*p* < 0.05), 55% (*p* < 0.01), 69.6% (*p* < 0.01), and 25.4% (*p* < 0.05) in the CdCl_2_ treatment group compared with the control group (Figure 4). Furthermore, the AST:ALT ratio in the CdSe/ZnS QDs treatment group and AST, AST:ALT ratio in CdCl_2_ treatment group exhibited a highly significant increase compared with the control group (Figure 4).

### 3.5. Histopathological Examination 

**Figure 5:**
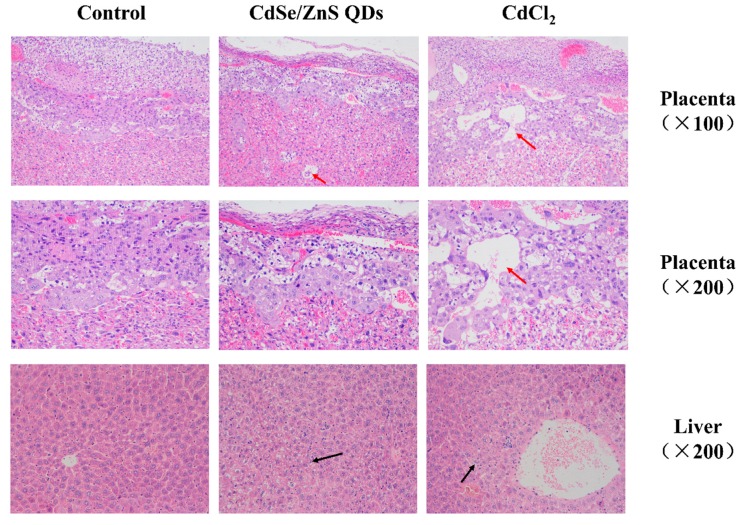
Histological images of the liver and placenta from GD18 P0 mice. Red arrows indicate the large vacuoles on placenta, black arrows indicate histopathological changes in the liver.

Histopathological changes in the liver and placenta of mice are shown in Figure 5. After CdSe/ZnS QDs or CdCl_2_ treatment, hepatocytes appeared swollen and vacuoles occurred in cytoplasm (Figure 5). Meanwhile, necrotic hepatocytes could be found in the CdCl_2_ group.

The histopathological changes in the placenta showed large vacuoles in the junction zone of the placenta of the CdSe/ZnS QDs and CdCl_2_ treatment groups (Figure 5). Sharp decline of blood cells was observed in the labyrinth zone. Meanwhile, some deformed trophoblast giant cells were found in the CdCl_2_ treatment group. 

### 3.6. Offspring Development

**Figure 6:**
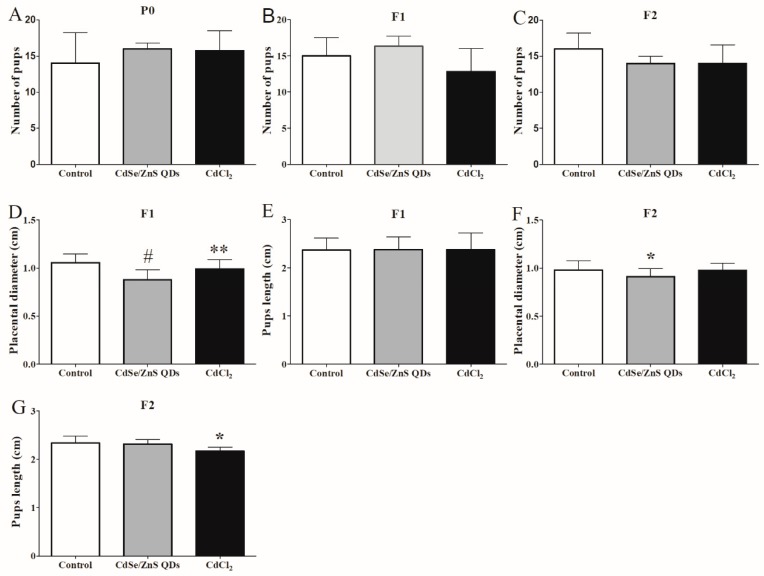
Reproductive ability and development status of mice in three generation after P0 exposure to QDs and CdCl_2_. (**A**) Number of pups in P0; (**B**) number of pups in F1; and (**C**) number of pups in F2. F1, F2 placental diameter (**D**,**E**) and fetus length (**F**,**G**). All the data are expressed as mean ± SD (n = 5). * *p* < 0.05, ** *p* < 0.01, # *p* < 0.001 vs. control.

**Figure 7:**
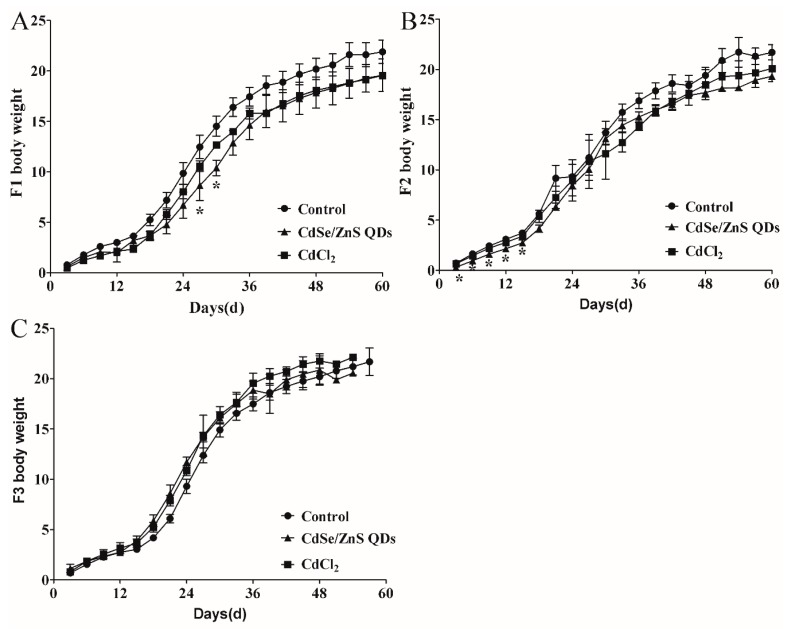
60-day body weight growth ratios: (**A**) F1 (n = 6); (**B**) F2 (n = 6); (**C**) F3 (n = 6). All the data are expressed as mean ± SD. * *p* < 0.05 vs. control. The body weight growth ratios = (bodyweight − body weight in PND1)/body weight in PND1.

There were no significant differences in the numbers of average litter between the treatment groups and control group of all the generations (Figure 6A–C). On GD 18, the fetal average body length in F1 did not show significant differences among all of the groups (Figure 6E). In addition, the average fetal body length in the CdCl_2_ treatment group of F2 was 2.17 cm at GD 18, which was significantly smaller than the control group (Figure 6G). The average diameter of the placenta in the CdSe/ZnS treatment group of F1 was 0.88 cm, which was 16.19% shorter than (1.05 cm) the control group (Figure 6D). The placental average diameter of F2 in CdSe/ZnS treatment group was 0.91 cm, which was also smaller than that of the control group (Figure 6F). The CdSe/ZnS and CdCl_2_ treatment groups (Figure 7A–C) exhibited a decrease in body weight among the P0’s, F1’s offspring F1, F2, respectively.

### 3.7. RT-qPCR Analysis

To study the mechanism of CdSe/ZnS QDs induced placenta injury and dysfunction, the genes associated with fetus development (*Ptch1, Smo, Gli1, Gli2, Gli3*), apoptosis (*Bcl-2,* and *Caspase3*), and oxidative stress (*HO-1, Gclc*) were chosen for RT-qPCR assay. The results of these studies were enumerated.

**Figure 8:**
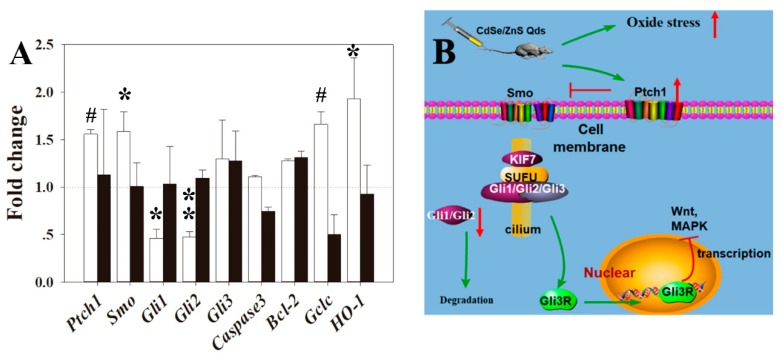
Gene expression and possible molecular mechanism after P0 exposure to QDs. (**A**) Fold changes of gene expression in P0 and F1 generations placentas. (**B**) Possible molecular mechanism. Relative gene expression levels were determined by the critical threshold (Ct) number and calculated using the 2^−ΔΔ*C*t^ method (fold change = 2^−ΔΔ*C*t^ value). Values were the mean ± standard deviation, n = 6. * *p* < 0.05, ** *p* < 0.01, # *p* < 0.001 vs. control.

In the P0 CdSe/ZnS QDs treatment group, *Ptch1, Smo, HO-1,* and *Gclc* were significantly upregulated in the placenta (Figure 8A). *Gli1* and *Gli2* in the placenta of P0 after CdSe/ZnS QDs treatment showed around a two-fold significant downregulation, respectively, while *Gli3* showed upregulation but it was not significant. No obvious change of these genes were found in the F1 CdSe/ZnS QDs treatment group.

## 4. Discussion

Due to the rapid growth and applications of nanotechnology, the likelihood of nanomaterials exposure to pregnant people could substantially increase. Nanoparticles may induce offspring injury in the following ways: (1) Pass through the placenta–fetus or blood–breast barrier and directly induce fetal injury [10]; (2) induce placenta or uterus injury and indirectly cause fetus injury [12,13]; or (3) induce maternal inflammation to induce fetal injury [14]. In this study, we evaluated the transgenerational effects and mechanism of action of CdSe/ZnS QDs toxicity in murine animals.

### 4.1. QDs Induced Maternal Injury

Hematology and serum biochemical indicators have been used to evaluate the alteration of physiological state of mice. WBC and PLT are responses of animals that may indicate system inflammation. A decrease in PLT could indicate inflammatory dysfunction in mice after CdSe/ZnS QDs treatment in GD 18. RBC, MCV, and HCT indices were also used to indicate possible anemia response. In this study, HCT and RBC levels showed significant decrease in PND 21 in the CdSe/ZnS QDs treatment, which implied that CdSe/ZnS QDs treatment induced anemia. These observations were consistent with previous research [12]. The Cr and TP, which may be used as indices of renal function, in the CdSe/ZnS QDs treatment group, suggesting possible kidney injury after treatment (Figure 4B). 

Histopathological examinations in the liver of CdSe/ZnS QDs and CdCl_2_ treatment groups exhibited swollen hepatocytes and vacuoles appears in cytoplasm, suggesting liver damage occurred (Figure 5). Large vacuoles were found in P0 CdSe/ZnS QDs and CdCl_2_ treatment groups’ placenta at the junction zone, which indicated that the CdSe/ZnS QDs affected the placental structure, which was similar to a previous study [15]. A sharp decrease in blood cells in the labyrinth zone and deformed trophoblast giant cells in the CdCl_2_ treatment groups were found. This implied that CdCl_2_ treatment may have disturbed the gas exchange and nutrient transportation function of the placenta in accordance with a previous study [15].

### 4.2. QDs Accumulate in the Placenta but Do Not Pass to the Next Generation

The dosage of QDs used in this experiment may have been insufficient to allow bigger quantity of Cd to cross through the placenta, as seen from the low quantity of Cd deposited in the fetus with a relative high concentration of Cd in the placenta of GD18. Meanwhile, QDs could accumulatively deposit in the placenta. Elevated Cd concentration in breast and blood was exhibited in treatment groups at PND 21 (P0) compared with the control (not significant). The concentration of Cd was higher in the breast than in the blood, reflecting the translocation and deposition of Cd in the breast. However, no obvious Cd concentration elevation in the stomach of PND 21 mice (F1) was found (data not shown). 

### 4.3. QDs Impaired the First Two Generations of Placenta Growth and Induced Offspring Injury

The offspring reproductive ability was evaluated by the number of weaned pups per litter. In this regard, no significant changes were found among all groups. Furthermore, there was no significant malformation of fetus found in the F1 and F2 generations. However, smaller placenta diameters were found in the CdSe/ZnS QDs treatment and CdCl_2_ groups in the F1 and F2 generation. Compared with the control group in F1 and F2, the average body weight of the treatment groups also showed a slight decrease, which indicated that CdSe/ZnS QDs or CdCl_2_ treatment might have caused offspring retardation in accordance with previous studies [9,13,16].

To explore the physiological and pathological status of the offspring, we performed hematological tests in the F1 generation. WBC count decreased in mice treated with CdCl_2_, which may reflect weakening of the immune system after heavy metal ion exposure [17]. The MCV was also decreased in the CdCl_2_ treatment group and MCH decreased in either the CdSe/ZnS QDs or CdCl_2_ treatment groups compared with the control group. In summary, the CdCl_2_ treatment caused more significant MCV and MCH level decrease than the CdSe/ZnS QDs treatment group. The significant decrease in MCV and MCH in mice exposed to CdSe/ZnS QDs and CdCl_2_ suggested the possibility of anemia in the F1 generation. Biochemical evaluation in F1 was carried out for AST:ALT ratio, ALP, and TBIL, which were used as reliable markers of liver inflammation [18]. In the F1 generation, AST level and AST:ALT ratio significantly increased in the CdSe/ZnS QDs treatment group, which indicated that there was serious liver damage induced by CdSe/ZnS QDs.

### 4.4. QD May Cause Toxicity through Shh Signal Pathway

The possible toxicity mechanism for the CdSe/ZnS QDs was examined through induction of offspring development retardation, transcriptional levels of genes related to development, apoptosis, and oxidative stress in the placenta. The Shh signal pathway has been known to play an important part in mammal pup’s development [19,20]. *Ptch1*, *Smo*, *Gli1*, *Gli2*, *Gli3* are the key genes in the Shh signal pathway and were chosen to reflect the status of the Shh pathway after treatment. Ptch1 is a transmembrane protein that acts as a receptor for sonic hedgehog (Shh). In this research, ptch1 showed upregulation, which implied that the shh signal pathway was in the off state. The Smo then transferred the signal into the cell and finally activated the Gli1 and Gli2, which acted as transcription activator. Although *Smo* was also upregulated, previous studies indicated that Ptch1 could efficiently inhibit Smo, and one Ptch1 molecule can inhibit 50 Smo molecules [21]. In the off state, Ptch1 bound with Smo, which inhibited Smo molecules causing the inactivation of the Gli1 and Gli2 [22]. When Gli1 and Gli2 are active, some downstream genes could be activated along with transcription, such as Wnt and MAPK, which are important in fetus development [23,24]. Gli3 acts as a negative transcription regulator in the Shh signal pathway “off” state, which could be a repressor form (GliR) that blocks transcription of downstream genes [25] (Figure 8A). Gli2 and Gli3 are required for the proper development of placenta with the Shh signal pathway [26]. In this study, the expression reduction of *Gli1* and *Gli2* were found in P0 after CdSe/ZnS QDs treatment, which indicated that those nanoparticles might have enhanced the shutting off the Shh signal pathway, which restricted placenta development. 

Many reports have indicated that nanoparticle toxicity was caused by ROS and apoptosis [27,28]. Caspase-3 is an important executor of cell apoptosis and apoptosis was thought to be followed with Caspase-3 expression [29]. It also reported that a member of the Bcl-2 family is important in regulating the process of cell death [30]. In this study, upregulation of *Caspase3* and *Bcl-2* were found in P0 but not significant. These implied that the CdSe/ZnS QDs treatments may not induce apoptosis in the placenta of P0. HO-1 and GCLC are related to oxidative stress [31,32]. HO-1 is a stress protein that is produced during oxidative challenges. Heme oxygenase mediates the first step of heme catabolism by cleaving the heme to form biliverdin that is transformed into bilirubin. Biliverdin has been shown to protect linoleic acid from oxidation [33]. On the other hand, GCLC is the first rate-limiting enzyme in GSH synthesis, which is an important organ antioxidant [34]. GSH can decrease ROS concentration that ultimately reduces the damage caused by ROS [35]. In this study, *HO-1* and *GCLC* were upregulated in P0 placenta, which indicated ROS stress in the F0 placenta. 

## 5. Conclusions

In this study, the maternal effect and three-generational development conditions after exposure to CdSe/ZnS QDs in perinatal stage have been studied. The results showed that exposure to CdCl_2_ and CdSe/ZnS QDs induced high accumulation of Cd in blood, breast, uterus, and placenta in the P0 generation. This accumulation of QDs induced maternal injury. Smaller placenta size and lower body weight growth rate in the first two generations and offspring (F1) injury were found after QDs exposure. The RT-qPCR results indicated that the toxic effects of CdSe/ZnS QDs might be through the shh signal pathway, and ROS stress may also be involved. Further studies are recommended to confirm the results obtained in this study to elucidate the QD mechanism of toxicity.

## Supplementary Materials

The following are available online at http://www.mdpi.com/2079-4991/9/2/257/s1, Figure S1: Serum biochemical analysis mice sacrificed at P0 GD 18 (A), PND 21 (B); Table S1: RT-qPCR primer pairs; Table S2: Whole blood analysis from P0 female mice treated with normal saline, CdSe/ZnS QDs, and CdCl_2_ in GD 18 stage; Table S3: Whole blood analysis from female mice treated with normal saline, CdSe/ZnS QDs, and CdCl_2_ in PND 21; Table S4: Whole blood analysis from F1 female mice treated with normal saline, CdSe/ZnS QDs and CdCl_2_.
