# Supplementary material for: CdSe/ZnS Quantum Dots Impaired the First Two Generations of Placenta Growth in an Animal Model, Based on the Shh Signaling Pathway
Source: Nanomaterials (Basel). 2019 Feb 14;9(2):257. doi: 10.3390/nano9020257 (PMC6409599; doi:10.3390/nano9020257)
Supplement: Supplementary file 1 [file nanomaterials-09-00257-s001.pdf]

Supplementary materials

CdSe/ZnS Quantum Dots Impaired the First Two Generations of Placenta Growth in an Animal Model, Based on the Shh Signaling Pathway

Wuding Hong [1], Huijuan Kuang [1], Xingping He [1], Lin Yang [1], Pengfei Yang [1], Bolu Chen [1], Zoraida P. Aguilar [2] and Hengyi Xu [1,*]

[1] State Key Laboratory of Food Science and Technology, Nanchang University, Nanchang 330047, China; 407205116020@email.ncu.edu.cn (W.H.); huijuankuang@126.com (H.K.); hxpoutlook@126.com (X.H.); ylwyh7729836@126.com (L.Y.); hnayypf@126.com (P.Y.); 407205117055@email.ncu.edu.cn (B.C.)

[2] Zystein, LLC., Fayetteville, AR 72704, USA; zaguilar@zystein.com

* Correspondence: kidyxu@163.com or HengyiXu@ncu.edu.cn; Tel.: +0086-791-8830-4447-ext-9520

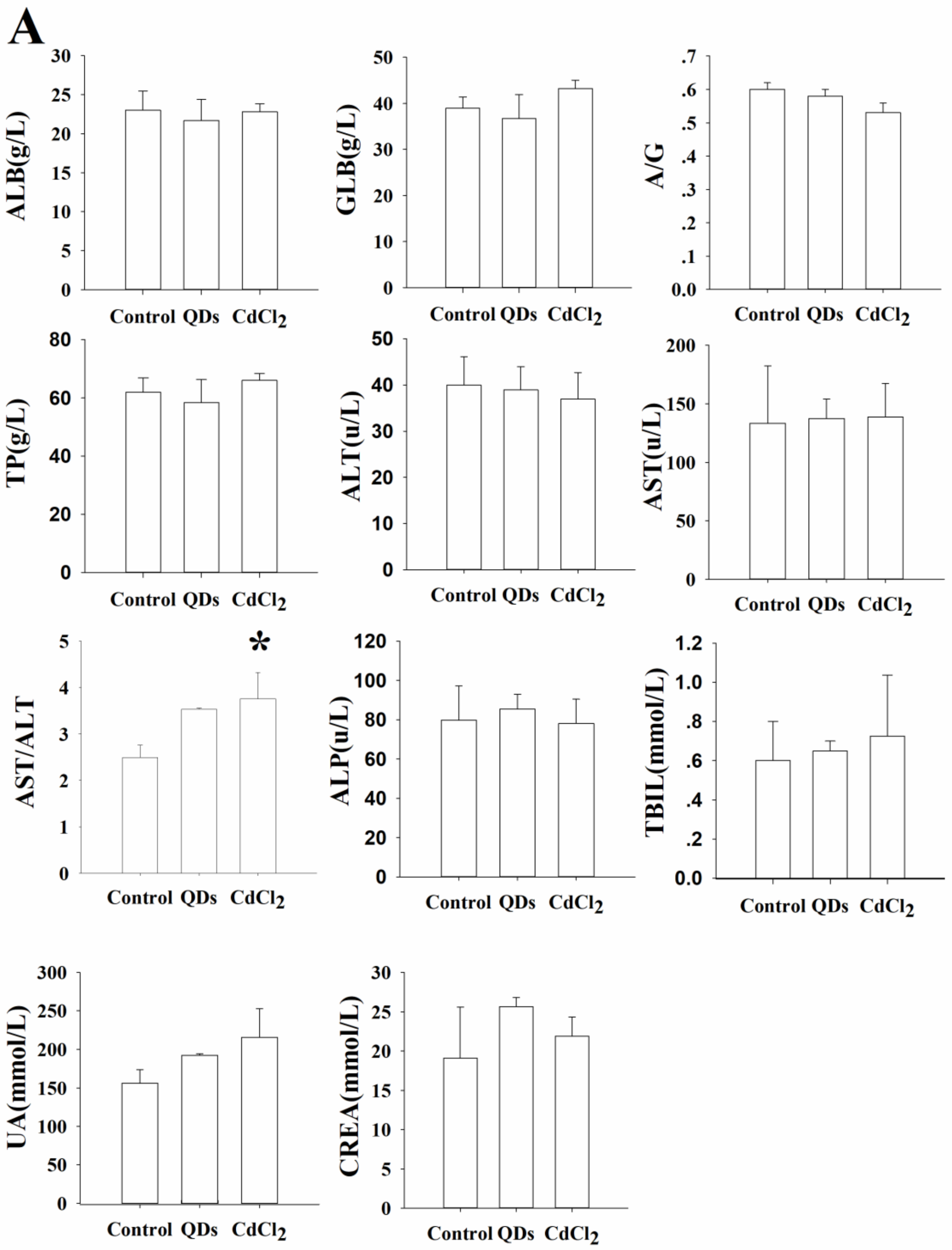
A
ALB(g/L)
GLB(g/L)
A/G
TP(g/L)
ALT(u/L)
AST(u/L)
AST/ALT
*
ALP(u/L)
TBIL(mmol/L)
UA(mmol/L)
CREA(mmol/L)
Control QDs CdCl2

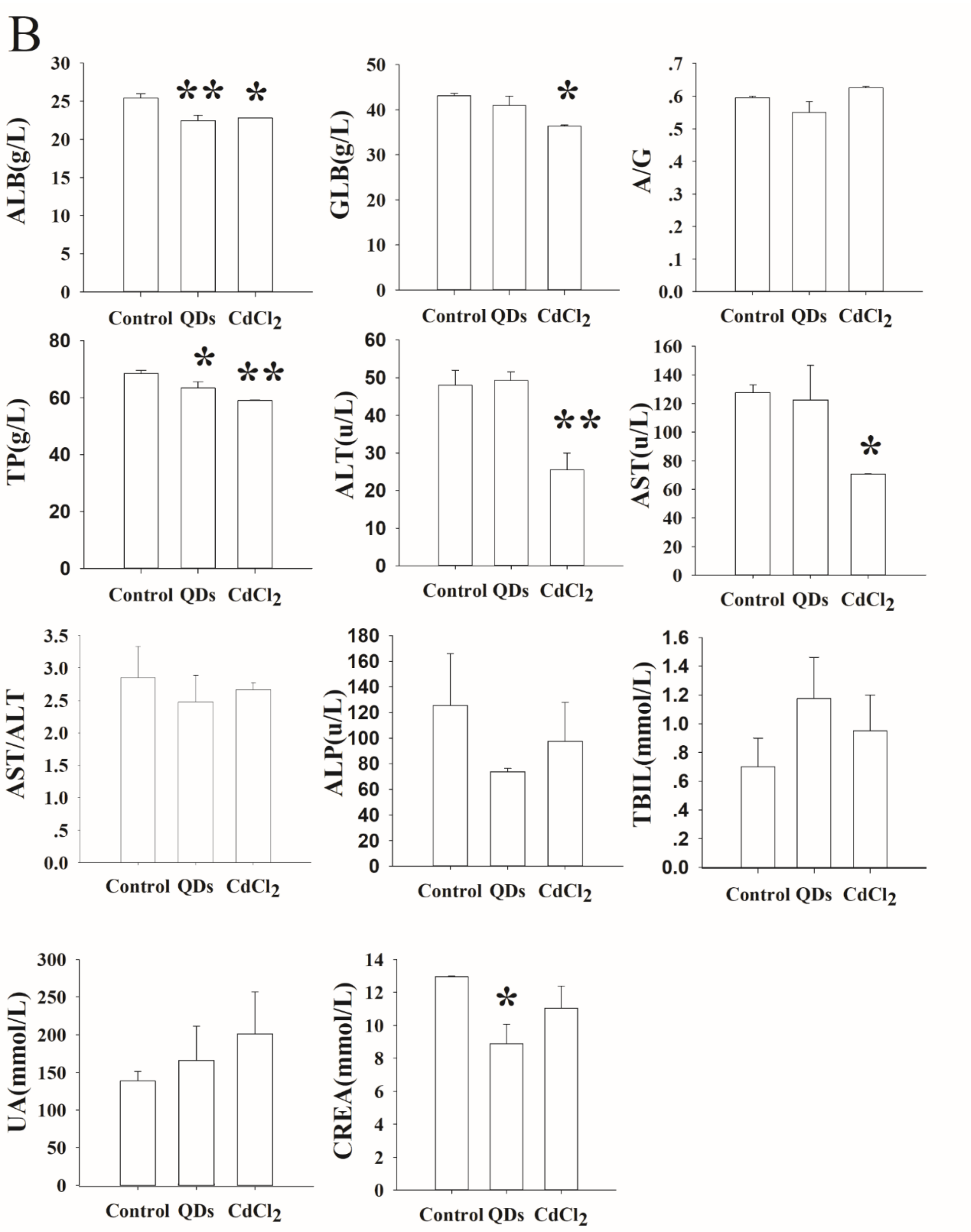

Figure S1. Serum biochemical analysis of mice sacrificed at P0 GD 18 (**A**); and PND 21 (**B**). *P < 0.05, **P < 0.01, #P< 0.001 vs. control.

Tables

Table S1. RT-qPCR primer pairs.

Gene	Description	Primer Sequence	Size (bp)
Smo	*Smo*-F	5'-CTTGATGGCTGGAGTAGTCTGG-3'	121
	Smo-R	5'-CGTGAGCAGGTGGAAATAGGA-3'	
SUFU	*SUFU*-F	5'-TTTCCTCCAGATTGTTGGTGTC-3'	97
	SUFU-R	5'-AATGGGCACTGTCCGTAGTAG-3'	
KIF7	*KIF7*-F	5'-CACCGTCTTTGCCTATGGTC-3'	151
	KIF7-R	5'-GTCCAGCAGGTCATTCTCATCA-3'	
Ptch1	*Ptch1*-F	5'-CCCGTCAGAAGATAGGAGAAG-3'	281
	Ptch1-R	5'-CCAGAAGCAGTCCAAAGGTG-3'	
Gli1	*Gli1*-F	5'-TACATGCTGGTGGTGCACAT-3'	162
	Gli1-R	5'-GCTGCAACCTTCTTGCTCAC-3'	
Gli2	*Gli2*-F	5'-GAAAGAAGCCAAGAGTGGTCTC-3'	236
	Gli2-R	5'-TGACAGGGCTGCCACTTAGG-3'	
Gli3	*Gli3*-F	5'-AGCAAGTGGTTCCTATGGGC-3'	173
	Gli3-R	5'-ATGTTGGAGCAGGGTGGATG-3'	
Caspase-3	*Caspase-3F*	5'-GGAGGCTGACTTCCTGTATGCTT-3'	157
	Caspase-3R	5'-CCTGTTAACGCGAGTGAGAATG-3'	
Bcl-2	*Bcl-2F*	5'-CACTCGACCTTGTTTCTTCCAG-3'	146
	Bcl-2R	5'-TCCTAACCCCTTGCTCTGCTT-3'	
Gclc	*Gclc*-F	5'-TGGCAGACAATGAGGTTT-3'	173
	Gclc-R	5'-AGCGGAATGAGGAAGTCT-3'	
HO-1	*HO-1*-F	5'-ACCGCCTTCCTGCTCAAC-3'	195
	HO-1-R	5'-GAGGAGCGGTGTCTGGGAT-3'	
GAPDH	*GAPDH*-F	5'-ATGTGTCCGTCGTGGATCTG-3'	242
	GAPDH-R	5'-GCCGTATTCATTGTCATACCAGG-3'	

Table S2. Whole blood analysis from P0 female mice treated with normal saline, CdSe/ZnS QDs, and $CdCl_2$ in GD 18 stage.

Whole Blood Parameter	Control	CdSe/ZnS QDs	$CdCl_2$
WBC × 10^9/L	9.43 ± 0.05	11.5 ± 1.3	12.5 *
RBC × 10^{12}/L	9.49 ± 1.34	9.19 ± 0.35	10.15
HGB	143.33 ± 19.26	131 ± 1.63	131.5 ± 12.5
HCT	0.51 ± 0.06	0.47 ± 0.02	0.47 ± 0.05
MCV	53.6 ± 1.19	51.57 ± 0.12 *	52.4 ± 0.4
MCH	15.1 ± 0.33	14.23 ± 0.41	14.6 ± 0.4
MCHC g/L	282 ± 6.16	279 ± 8.6	278.5 ± 5.5
PLT × 10^9/L	1357.5 ± 275.5	700 ± 61 *	1509.5 ± 70.5

Values are expressed as mean ± SD, n = 4 in each group.

*P < 0.05, **P < 0.01 vs. control.

Table S3. Whole blood analysis from female mice treated with normal saline, CdSe/ZnS QDs, and $CdCl_2$ in PND 21.

**Whole Blood Parameter**	**Control**	**CdSe/ZnS QDs**	**$CdCl_2$**
WBC × 10^9/L	7.7 ± 0.9	11.3 ± 1.76	8.65 ± 1.45
RBC × 10^{12}/L	12.41 ± 0.65	10.31 ± 0.87 *	10.04 ± 0.37 *
HGB	173 ± 4	148.25 ± 7.73	101 ± 46
HCT	0.65 ± 0.03	0.53 ± 0.04 *	0.57 ± 0.03
MCV	52.55 ± 0.05	51.05 ± 1.82	56.8 ± 0.4 *
MCH	14 ± 0.4	14.23 ± 0.82	15.05 ± 0.15
MCHC g/L	265.5 ± 7.5	283 ± 17.76	265.5 ± 4.5
PLT × 10^9/L	1585 ± 327	1395.75 ± 198.93	1298 ± 62

Values are expressed as mean ± SD, n = 4 in each group.

*$P < 0.05$, **$P < 0.01$ vs. control.

Table S4. Whole blood analysis from F1 female mice treated with normal saline, CdSe/ZnS QDs, and $CdCl_2$.

Whole Blood Parameter	Control	CdSe/ZnS QDs	$CdCl_2$
WBC × 10^9/L	8.03 ± 0.97	7.48 ± 0.89	5.2 ± 1.56 **
RBC × 10^{12}/L	8.42 ± 0.59	9.31 ± 0.59	8.53 ± 0.51
HGB	165 ± 13.55	168.25 ± 6.68	149.338 ± 11.00
HCT	0.56 ± 0.07	0.62 ± 0.04	0.51 ± 0.05
MCV	65.98 ± 3.93	66.2 ± 3.57	59.68 ± 3.25 *
MCH	19.58 ± 0.33	17.93 ± 0.48 *	17.52 ± 0.99 **
MCHC g/L	297.75 ± 13.25	273.75 ± 14.04	294.33 ± 18.50
PLT × 10^9/L	905.67 ± 135.22	667.5 ± 109.37	826.67 ± 301.74

Values are expressed as mean ± SD, n = 4 in each group.

*P < 0.05, **P < 0.01 vs. control.
